# Towards a Sampling Rationale for African Swine Fever Virus Detection in Pork Products

**DOI:** 10.3390/foods9091148

**Published:** 2020-08-20

**Authors:** John Flannery, Rebecca Moore, Laura Marsella, Katie Harris, Martin Ashby, Paulina Rajko-Nenow, Helen Roberts, Simon Gubbins, Carrie Batten

**Affiliations:** 1 The Pirbright Institute, Ash Road, Pirbright GU24 0NF, UK; rebecca.moore@pirbright.ac.uk (R.M.); Laura.marsella@pirbright.ac.uk (L.M.); katie.harris@pirbright.ac.uk (K.H.); martin.ashby@pirbright.ac.uk (M.A.); paulina.rajko-nenow@pirbright.ac.uk (P.R.-N.); simon.gubbins@pirbright.ac.uk (S.G.); carrie.batten@pirbright.ac.uk (C.B.); 2 Defra, Nobel House, 17 Smith Square, London SW1P 3JR, UK; helen.roberts@defra.gov.uk

**Keywords:** African swine fever virus, qPCR, surveillance, food testing, sausage

## Abstract

African swine fever (ASF) is a highly lethal disease of pigs caused by the ASF virus (ASFV), which presents a serious threat to global food security. The movement of contaminated pork products has previously been postulated as contributing to the introduction of ASF into new areas. To evaluate the performance of ASFV detection systems in multi-component pork products, we spiked sausage meat with four different ASFV-containing materials (ASFV cell culture, pork loin, meat juice and bone marrow). DNA was extracted using two manual systems (MagMAX CORE, Qiagen) and one automated (MagMAX CORE) one, and three qPCR assays (VetMAX, King, UPL) were used. The performance of the DNA extraction systems was as follows; automated MagMAX > manual MagMAX > manual Qiagen. The commercial VetMAX qPCR assay yielded significantly lower C_T_ values (*p* < 0.001), showing greater sensitivity than the World Organization for Animal Health (OIE)-prescribed assays (King, UPL). Detection probability was the highest for matrices contaminated with bone marrow compared with pork loin or meat juice. An estimated minimum sample size of one 1-g sample is sufficient to detect ASFV in a homogenous pork product if bone marrow from infected pigs comprises 1 part in 10,000. We demonstrated that existing ASFV detection systems are appropriate for use in a food-testing capacity, which can provide an additional control measure for ASF.

## 1. Introduction

African Swine Fever (ASF) is a highly lethal viral disease of pigs caused by the ASF virus (ASFV), a large DNA virus of the *Asfarviridae* family [1]. The virus was first described in 1921 in Kenya and was subsequently found throughout sub-Saharan Africa, where it is maintained through an infection cycle between ticks and wild suid populations [2]. ASFV does not cause disease in humans, and there is no public health risk. However, due to its serious socioeconomic consequences and importance for international trade of live pigs and pork, ASF is a World Organization for Animal Health (OIE) notifiable disease. ASF most often presents in non-endemic countries as a peracute or acute disease 3–4 days post-infection, with initial clinical signs which include high fever, loss of appetite and lethargy [3]. The disease progresses to cause skin erythema, pulmonary oedema, hyperaemic splenomegaly and petechial haemorrhages throughout internal organs. Death can occur within 1-week post-onset of clinical signs. ASFV is most commonly transmitted directly between pigs through oronasal contact or indirectly through contact with feed or fomites contaminated with the virus [4]. However, the human-mediated spread of ASFV has played a critical role in the global epidemiology of the disease. The first incidence of ASF outside Africa occurred in Portugal in 1957 and was caused by ASFV-contaminated food products being fed to pigs [3], which resulted in the dissemination of the virus throughout the Iberian Peninsula. Since that time, numerous outbreaks of ASF have been caused by catering waste being fed to pigs [5]. The most significant incursion of ASFV occurred in Georgia in 2007 from East Africa, introduced by international catering waste, which contained ASFV-contaminated meat, at the port of Poti [6] and has caused ASF to spread throughout Europe and Asia posing a considerable threat to global food security. For instance, estimates suggest 150–200 million pigs (approximately 30% of the Chinese pig population) had been lost to ASF by mid-2019, although some reports suggest that this could be as great as 50–70% [7]. This decline in pork caused a concurrent 50% increase in pork prices in China during 2019 [8]. Although a number of ASF vaccine candidates have been described [9,10,11], none have been licensed for use in the European Union (EU), and control relies on international cooperation, enhanced biosecurity and appropriate surveillance strategies.

A European Food Safety Authority (EFSA) report from 2010 considered there to be a moderate likelihood of ASF becoming established in the EU in the wild boar population, but the risk was considered to be low for commercial farms [12]. This has generally been the case, but perhaps more importantly, the report considered a high likelihood of further spread in low-biosecurity, non-commercial settings, as found in much of Eastern Europe. In these low biosecurity settings, the main risk routes were considered to be in contact with infected wild boar and the use of swill feed. Indeed numerous reports of ASF outbreaks have been described in these settings in eastern Europe [13]. There have been no reports of ASF incursions in domestic pigs in the EU via the swill-feeding pathway since the introduction of an EU ban in 2001, although it could not be excluded in some outbreaks in Poland [14]. Boar-mediated spread has largely caused the disease to expand throughout Europe [15] and Russia [16]. In 2018, ASF was detected in wild boar in Belgium, which represented a large geographic jump, most likely to be human-mediated by the transportation of contaminated products or fomites to an area with a high population of wild boar [17].

To ensure that countries affected by ASF can continue to trade within the EU, a regionalised approach has been devised as outlined in the Commission Implementing Decision (EU) EU 2020/543. Briefly, the export of pork meat from areas within a restricted zone is prohibited [18]; however, fresh or frozen pork meat can continue to be traded from areas outside these zones. While surveillance programmes have been implemented in a number of affected EU member states, the possibility exists that contaminated meat could be traded in good faith prior to the detection of ASFV within an area. This has previously occurred in the United Kingdom (UK), following the legal importation of meat from Romania in 2018 [19], resulting in a lengthy tracing exercise with the Romanian authorities which allowed the risk to be mitigated without testing the products.

EU animal health regulations stipulate that ASF diagnosis is confirmed using two independent virus detection methods [20]. Real-time PCR (qPCR) is the most sensitive and widely used diagnostic method, and prescribed assays exist. ASFV causes a high viremia (up to 10^9^ TCID_50_ mL^−1^ blood) [21], and it is readily detectable in a variety of sample matrices (EDTA blood and tissues, such as lymph nodes, spleen, lung and kidney) [22]. Thus the chances of detecting ASFV in food products are quite high. Although the UK meat products’ regulations prohibit the use of brains, lungs, spleen and stomach amongst other internal organs in meat products [23], blood is present in many products, the intestine can be used as sausage skin, and minced meat can contain bone fragments. Sausage meat is a multi-component food product which consists of salt, spices, binders, meal and can comprise meat from numerous animals, either from cuts of meat or from mechanically separated meat. Considering the potential for numerous pigs to contribute to a multi-component meat product, such as sausage meat, it could provide a suitable sampling matrix for surveillance purposes. Clearly, testing of pork-containing products for human consumption would be important to either prevent ASFV-contaminated products from entering the food chain or to assure freedom before export. We, therefore, aimed to determine whether the most-widely utilised nucleic acid extraction systems and qPCR assays (collectively referred to as ASFV detection systems) could be useful in a food testing capacity to detect ASFV. By creating ASFV-contaminated food matrices, we aimed to determine the comparative analytical sensitivity of these ASFV detection systems and extrapolate their usefulness in testing a multi-component food product.

## 2. Materials and Methods

### 2.1. Preparation of Samples for Evaluation

**Figure 1:**
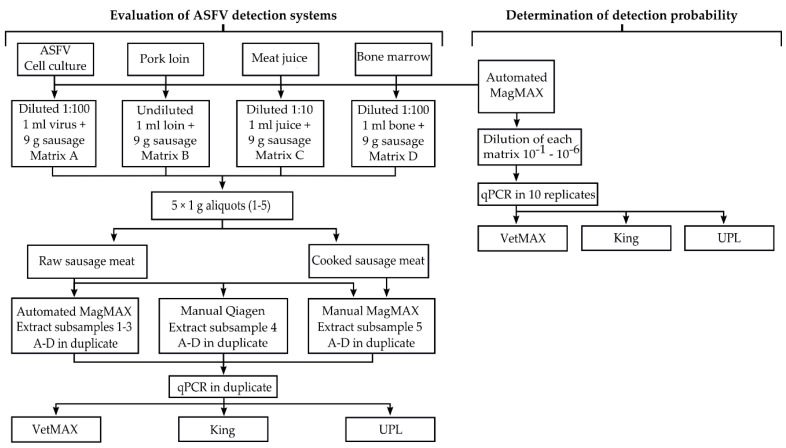
Flowchart of the evaluation of African swine fever virus (ASFV) detection systems.

Figure 1 shows the experimental plan to evaluate the ASFV detection systems. Sausage meat containing >72% pork meat was purchased from a UK retail supermarket and was spiked with ASFV-containing material to create four different testing matrices (A–D). Matrix A was spiked with an ASFV genotype II cell culture isolate obtained from the first outbreak of ASF in Hong Kong in December 2019. Matrices B–D, respectively, were spiked with pork loin, meat juice and bone marrow, obtained from a pig that was experimentally-infected with ASFV genotype II (Georgia 2007/1). This experiment was approved by the Animal Welfare and Ethical Review Board of The Pirbright Institute in compliance with a national Project License (number 70/8852) granted by the UK Home Office. This animal showed moderate clinical signs of ASF, such as respiratory distress, lethargy, inappettence, pyrexia (>41 °C) and haemorrhagic areas on its ears and was euthanized at 6 days post-infection having reached the humane endpoint. In our remit as OIE reference laboratory for ASF, we have previously detected ASFV in a number of ASFV-contaminated food products at C_T_ values ranging between 34 and 38. Therefore, we prepared appropriate dilutions of this material, as indicated in Figure 1, using sterile PBS to create representative ASFV-contaminated food products. Approximately 10 g of the contaminated matrices (A–D) were prepared, and following manual homogenization using a spatula, the material was distributed equally to 10 tubes and was stored at −80 °C until processing.

### 2.2. Processing of Meat Samples—Homogenisation

Approximately 1 g sausage meat from each testing matrix (A–D) was thawed and homogenised using sterile sand and 5 mL PBS with a mortar and pestle. The suspension then was centrifuged at 3000× *g* for 5 min, and the resulting supernatant was removed for further testing. The supernatant was stored at 4 °C until DNA extraction (within 24 h).

### 2.3. Extraction of ASFV DNA

Three different nucleic acid extraction systems were used on the homogenates: an automated extraction and two manual extraction systems in accordance with the manufacturer’s instructions. Two hundred microlitres homogenate supernatant was extracted in duplicate using the MagMAX™ CORE Nucleic Acid Purification Kit reagents (ThermoFisher Scientific, Paisley, UK) (hereafter MagMAX) on the automated extraction platform; the KingFisher Flex Purification System (ThermoFisher Scientific, Paisley, UK). Two hundred micorlitres homogenate supernatant was extracted in duplicate using the MagMAX manually using a magnetic rack and aspiration with a pipette. For both automated and manual MagMAX CORE-extractions, ASFV DNA was eluted into a 90 µL elution buffer. The third extraction system was the QiAmp Viral Mini RNA kit (Qiagen, Manchester, UK) (hereafter Qiagen), where 140 µL homogenate supernatant was extracted manually in duplicate, and ASFV DNA was eluted into a 50 µL AVE buffer. DNA extracts were stored at −20 °C before further analysis.

### 2.4. ASFV qPCR Assays

DNA extracts were analysed in duplicate using three ASFV qPCR assays. The commercially-available VetMAX™ African Swine Fever Virus Detection Kit (ThermoFisher Scientific, Paisley, UK) (hereafter VetMAX) was performed as per the manufacturer’s instructions using 5 µL DNA. Using the VetMAX, ASFV was detected using the FAM channel, while the internal positive control (IPC) was detected using the VIC channel of the instrument. We also investigated two OIE-prescribed qPCR assays which target different regions within ASFV VP72. The assays described by King et al., 2003 [24] (hereafter King) and Fernandez-Pinero et al., 2013 [25] (hereafter UPL) were performed on 2 µL of DNA using the recommended primer/probe concentration and the Path-ID™ qPCR Master Mix (ThermoFisher Scientific, Paisley, UK). All assays were performed on an Applied Biosystems 7500 fast instrument (ThermoFisher Scientific, Paisley, UK).

### 2.5. Evaluation of the ASFV Detection Systems

From each of the testing matrices A–D, three aliquots were extracted in duplicate using the automated KingFisher Flex system. For the manual extraction systems, DNA was extracted in duplicate on one aliquot of each testing matrix. To simulate the cooking process, an additional aliquot of each matrix was heated at 76 °C for 15 min in a heating block and was manually extracted in duplicate using the MagMAX CORE. Finally, to determine the sensitivity of the three qPCR assays, serial dilutions (10^−1^ to 10^−6^) of DNA extracted from the original spiking material (used to create matrices A–D) were prepared using PBS. Each dilution was analysed in 10 replicates using the three ASFV qPCR assays, as indicated in Figure 1.

### 2.6. Statistical Analysis

To assess the performance of the ASFV detection systems, ASFV qPCR C_T_ values were analysed using a linear model. More specifically, the C_T_ value was the response variable, while matrices A–D (containing sausage and ASFV isolate, pork loin, meat juice or bone marrow) and assay (King, UPL or VetMAX) were explanatory variables. Model selection proceeded by stepwise deletion of non-significant (*p* > 0.05) terms, starting from a model including matrix, assay, and an interaction between them.

Using the C_T_ values from the dilutions of spiking material, the probability of a positive sample was estimated by fitting a binomial family generalised linear model with a logit link function to the test data. The proportion of tests positive for ASFV DNA (defined as any C_T_ value) was the response variable with material, dilution and assay as explanatory variables. Model selection proceeded by stepwise deletion of non-significant (*p* > 0.05) terms, starting from a model including material, assay and dilution. To determine appropriate sample sizes with 95% confidence of detecting ASFV, the following formula was applied:

log_10_(0.05)/log_10_(1 − (*p*/100)) (1)

where *p* is the probability (as %) of detection. Analyses were implemented in R version 3.6.1 [26].

## 3. Results

### 3.1. Performance of ASFV Detection Systems on Raw Food Matrices

ASFV DNA was detected in all sample matrices using the three different DNA extraction systems and qPCR assays. ASFV C_T_ values achieved by qPCR assays following automated extraction of sample matrices A–D are shown in Table 1.

**Figure 2:**
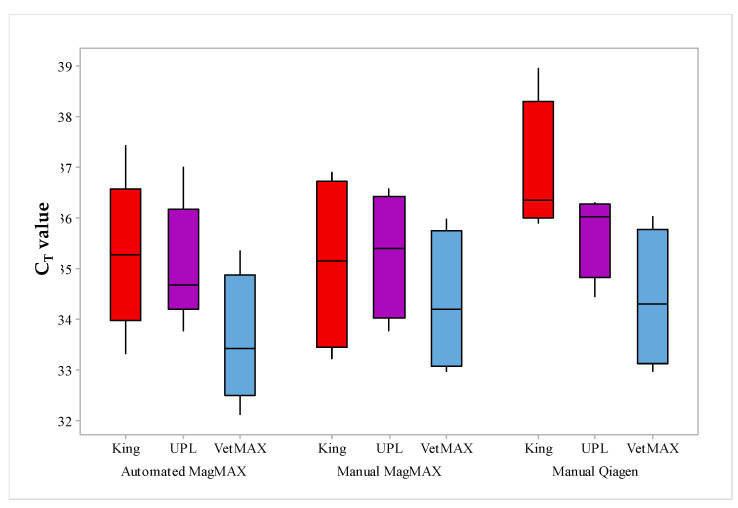
Boxplot of C_T_ values obtained following automated or manual extraction systems. The median C_T_ value and the interquartile range for each assay following each extraction system is shown.

Across each matrix, the mean C_T_ value achieved by the VetMAX was 33.66 in comparison with the mean C_T_ for the King (35.29) and UPL (35.08) assays. The mean difference between the VetMAX qPCR and the King and UPL assays was −1.62 and −1.41 C_T_ values, respectively. This was statistically significant using a one-way ANOVA (*p* < 0.044). Figure 2 shows a boxplot of C_T_ values for all three qPCR assays following automated or manual extraction. Across all matrices tested and all qPCR assays, the automated extraction system provided lower C_T_ values than the manual MagMAX (0.25 C_T_ values) and Qiagen (0.92 C_T_ values) extraction systems. C_T_ values for all qPCR assays were strongly correlated (*r* ≥ 0.934) between the automated and manual MagMAX extracted DNA. Considering the manual extraction systems, the MagMAX yielded lower mean C_T_ values than the Qiagen for each of the qPCR assays: VetMAX: −0.54 C_T_ values; King: −1.69 C_T_ values, and UPL: −0.42 C_T_ values. In summary, the performance of the extraction systems was automated MagMAX > manual MagMAX > manual Qiagen.

### 3.2. Performance of ASFV Detection System on Cooked Food Matrices

Table 2 shows the ASFV C_T_ values obtained in the heat-treated aliquots. Although C_T_ values were higher in the cooked matrices than the raw matrices, this was not found to be significant (*p* > 0.130). The VetMAX yielded lower C_T_ values than the King or UPL assays. However, this was not found to be significant using a one-way ANOVA (*p* = 0.34). The King assay did not detect ASFV in the heat-treated testing matrix C. The mean C_T_ value for the VetMAX internal positive control in cooked matrices was significantly higher (one-way ANOVA, *p* < 0.001) than raw matrices indicating that PCR inhibition was introduced during the cooking process.

### 3.3. Detection Probability of ASFV qPCR Assays

DNA extracted from the neat spiking material was diluted serially to 10^−6^ and was analysed to compare the analytical sensitivity of the three qPCR assays. This was also performed to approximate a situation where a single infected pig could be processed within a multi-component pork product and to determine the detection probability. The probability of detection decreased with an increasing dilution of ASFV-containing material (Table 3). The VetMAX assay showed the greatest probability of detection compared with both the King and UPL assays (which did not differ from one another).

There was no significant interaction between spiking material and assay (*p* = 0.07), but C_T_ values differed significantly (*p* < 0.001) amongst materials and assays. C_T_ values did not differ significantly (*p* = 0.98) between ASFV cell culture isolate and bone marrow but were significantly (*p* < 0.001) lower (~2 C_T_ values) than in pork loin or meat juice. C_T_ values did not differ significantly (*p* = 0.60) between pork loin and meat juice. The VetMAX yielded significantly lower C_T_ values (*p* < 0.001) than the King or UPL assays, which did not differ significantly between themselves (*p* = 0.51). The probability of a positive test result depended significantly (each *p* < 0.001) on the dilution of the sample, the material and the assay (Table 3). The probability of a positive test result decreased with the increased dilution of the sample. The probability of detection was greater for the VetMAX assay than the King and UPL assays (which did not differ significantly from one another). Considering the more-representative spiking materials we used, the detection probability was highest for bone marrow, then meat juice and was lowest for pork loin.

Using the detection probabilities, we calculated the minimum sample size to detect a single ASFV-positive animal within a homogenous pork product (Table 4). As pork loin contained the lowest concentration of ASFV in comparison with meat juice or bone marrow, a larger minimum sample size would be required to give confidence in detecting ASFV. In general, this indicates that ASFV can be reliably detected using a minimum sample size of 1 (i.e., one 1-g sample) in a homogenous product when the ASFV-contaminated material comprises 1 part in a hundred. Using a sample size of 1, ASFV can be detected in a homogenous food product where bone marrow comprises 1 part in 10,000. The different analytical sensitivity shown for the three ASFV qPCR assays impacted the calculated minimum sample sizes. For instance, when sampling a pork product containing ASFV-contaminated pork loin comprising 1 part in 10,000, the minimum sample size when using the VetMAX (*n* = 7) was much less than that for the UPL (*n* = 44) or King (*n* = 69) assays.

## 4. Discussion

Due to the increasing human population, global meat consumption has increased to meet demand. Pigs provide a source of inexpensive, high-quality animal protein because of their efficient feed conversion, high fertility and quick turnover. Indeed, pork is now the most-consumed terrestrial meat in the EU and accounts for over 36% of global meat intake [27]. ASF presents a serious risk to animal health and food security. The continued global spread of ASF is due to the lack of a vaccine, the stability of ASFV in the environment and in infected pork [28] and shortfalls in biosecurity measures [15]. Apart from boar-mediated transmission, the main pathways of introduction to the UK (and other ASF-free countries) are through ASFV-contaminated food products or fomites, illegally imported infected pigs or pork products thereof, or through legal imports from countries with as-yet undetected ASF [15]. Veterinarians provide a critical control point in the food chain where an organoleptic assessment is carried out by the veterinarian before slaughter [29]. Thus, considering the gross lesions caused by ASFV, it is likely that an affected animal would be identified at this point. However, the misdiagnosis of ASF as contagious porcine pleuropneumonia was identified as a cause of an outbreak at a commercial farm in China [30], and there is increasing evidence that in areas with endemic ASF, pathogenicity and, therefore, clinical detection may be reduced [31].

Assurance that the existing testing methodologies can detect ASFV in food matrices is important since contaminated foodstuffs can pose a source of infection for pigs if not properly discarded (as evidenced in the ASFV outbreak which occurred in Belgium in 2018) [17]. To approximate the detection of a single-infected animal within a batch of sausage meat, we spiked sausage meat with ASFV to levels comparable with those which we have detected in contaminated pork products. Using multiple-replicate analysis of serially-diluted ASFV-contaminated meat, we assessed the performance of different ASFV detection systems. All DNA extraction systems were suitable to yield detectable ASFV, though the automated MagMAX provided the best sensitivity. Nevertheless, we found that the manual MagMAX extraction protocol was appropriate to yield comparable C_T_ values to those using the automated system. The commercial VetMAX assay showed the greatest sensitivity and had a higher probability of detecting the virus in contaminated pork products in comparison with the OIE-prescribed assays (King and UPL). Although the King and UPL assays are widely used and are indeed suitable for the detection of ASFV in a diagnostic capacity, the routine testing of foodstuffs will carry an additional cost, and it is in this instance that assay performance can have an impact on the number of samples to be taken. For instance, when sampling a pork product containing ASFV-contaminated pork loin comprising 1 part in 10,000, the minimum sample size when using the VetMAX (*n* = 7) was much less than that for the UPL (*n* = 44) or King (*n* = 69) assays. Therefore, when considering the testing of pork products for ASFV, the performance of the available assays should be determined and then factored into the sampling rationale. While we aimed to determine the minimum sample size (number of 1-g samples) to detect ASFV, the specific contribution of an individual animal per multi-component food product (i.e., sausage meat) could not be established. Nevertheless, our study is a preliminary step towards developing a sampling rationale, and our findings should be considered as a conservative estimate of a suitable sample size.

The testing of pork products, in conjunction with animal health surveillance programmes, could be important to prevent ASFV-contaminated products from entering the food chain and thus being diverted through food waste to animal feed. ASFV DNA has been detected in seizures of pork meat at border inspections in Taiwan [32], Republic of Korea [33], Northern Ireland [34] and Australia [35]. Therefore, the sampling of pork-products at border control points could provide an essential control strategy to prevent such an incursion of disease. The manual MagMAX extraction could be useful in such a situation as it detected ASFV in cooked food products, as it does not require specialised equipment and DNA extraction can be performed quickly (approximately 10 samples within 20 min). By incorporating this relatively simple DNA extraction technology with a rapid ASFV detection assay, such as loop-mediated isothermal amplification [36], the reliable detection of ASFV could be performed in a relatively short period (<40 min) without the need for expensive instrumentation. This could allow for rapid screening at border control points or in regional laboratories—with positive results being subsequently confirmed in a national reference laboratory.

The EU regionalised approach has allowed the trade of pork products to continue, but given the ongoing spread of ASF into new regions throughout Europe, it may be appropriate to instigate sampling programmes in meat processing plants or at border inspection points on pork products originating from ASF-free regions. In the EU, food is tested for bacterial pathogens, such as *Salmonella* spp. and *Listeria* spp., to address human health concerns [37]. In general, the testing of foodstuffs for viruses is not widely practiced: One exception being the testing of bivalve molluscs which are eaten raw or lightly cooked. There exists the potential for ASFV testing to address a potential shortfall in the ability to detect viruses that pose a risk to animal health. A standardised method has been developed for the detection of norovirus and hepatitis A virus in foodstuffs [38], it may be prudent to consider a similar approach for viruses which cause notifiable veterinary diseases of high concern, such as ASFV.

The global reliance on pork products and the risk of ASF to food sustainability presents a serious concern for animal and food health authorities. It is, therefore, vital that efforts to control ASF, are maintained and, where possible, strengthened to ensure food security. The testing of food products provides a useful means to ensure that contaminated products do not enter the food chain with subsequent financial and animal health impacts. Coupled with animal health surveillance programmes, this can support freedom from disease within the national herd.

## Figures and Tables

**Table 1:** C_T_ values generated using automated extraction for all sample matrices.

Testing Matrix and Mean C_T_ Value (Range)					
**qPCR Assay**	**A** **(Sausage + Cell Culture Isolate)**	**B** **(Sausage + Pork Loin)**	**C** **(Sausage + Meat Juice)**	**D** **(Sausage + Bone Marrow)**	**Mean C_T_**
**VetMAX**	32.48 (32.43–32.52)	34.63 (34.13–34.90)	35.03 (34.59–35.37)	32.52 (32.17–32.73)	33.66
**King**	33.82 (33.26–34.34)	36.23 (35.24–36.71)	36.86 (35.86–37.43)	34.23 (33.64–34.74)	35.29
**UPL**	34.20 (34.19–34.21)	36.44 (35.41–37.01)	35.66 (35.09–36.45)	34.01 (33.78–34.25)	35.08

**Table 2:** C_T_ values obtained in cooked meat products using manual MagMAX extraction.

	Testing Matrix and Mean C_T_ Value (Range)			
	**A** **(Sausage + Cell Culture Isolate)**	**B** **(Sausage + Pork Loin)**	**C** **(Sausage + Meat Juice)**	**D** **(Sausage + Bone Marrow)**
VetMAX	35.85 (35.56–36.14)	37.12 (36.43–37.80)	36.80 (36.43–37.10)	34.25 (34.22–34.28)
King	36.24 (36.10–36.37)	36.84 (n.d.)	Undet. (n.d.)	35.49 (35.39–35.60)
UPL	37.44 (37.23–37.66)	37.60 (37.22–37.98)	36.40 (35.76–37.04)	35.49 (35.41–35.57)

Undet. undetected using qPCR, n.d. not determined due to insufficient data.

**Table 3:** Estimated probability (%) of a positive test result for each assay, material and dilution.

Spiking Material	Assay (Neat C_T_ Value)	Detection Probability % at Each log_10_ Dilution					
		−1	−2	−3	−4	−5	−6
**ASFV isolate**	King (18.91)	100	100	100	99.9	95.8	42.5
	UPL (17.71)	100	100	100	99.9	97.3	53.8
	VetMAX (18.98)	100	100	100	100	99.7	90.9
**Pork loin**	King (28.77)	99.9	97.7	57.9	4.3	0.1	0
	UPL (26.78)	100	98.5	68.4	6.6	0.2	0
	VetMAX (28.28)	100	99.8	94.9	37.7	1.9	0.1
**Meat juice**	King (23.79)	100	100	98.8	72.6	8	0.3
	UPL (22.59)	100	100	99.2	80.7	12	0.4
	VetMAX (23.83)	100	100	99.9	97.3	53.9	3.7
**Bone marrow**	King (20.71)	100	100	100	98.8	72.6	8
	UPL (19.42)	100	100	100	99.2	80.7	12
	VetMAX (20.49)	100	100	100	99.9	97.3	53.9

**Table 4:** Minimum sample size to detect a single infected animal per homogenous product.

Spiking Material	Assay	Log Diluted Component and Minimum Sample Size for Detection (95% CI)					
		**−1**	**−2**	**−3**	**−4**	**−5**	**−6**
**Pork loin**	King	1	1	4	69	2995	n.d.
	UPL	1	1	3	44	1497	n.d.
	VetMAX	1	1	2	7	157	2995
**Meat juice**	King	1	1	1	3	36	998
	UP	1	1	1	2	24	748
	VetMAX	1	1	1	1	4	80
**Bone marrow**	King	1	1	1	1	3	36
	UPL	1	1	1	1	2	24
	VetMAX	1	1	1	1	1	4

n.d. not determined due to insufficient data.
